# Methotrexate-Related Response on Human Peripheral Blood Mononuclear Cells May Be Modulated by the Ala16Val-SOD2 Gene Polymorphism

**DOI:** 10.1371/journal.pone.0107299

**Published:** 2014-10-20

**Authors:** Fernanda Barbisan, Jéssica de Rosso Motta, Alexis Trott, Verônica Azzolin, Eduardo Bortoluzzi Dornelles, Matheus Marcon, Thaís Doeler Algarve, Marta Maria Medeiros Frescura Duarte, Clarice Pinheiro Mostardeiro, Taís Cristina Unfer, Karen Lilian Schott, Ivana Beatrice Mânica da Cruz

**Affiliations:** 1 Biogenomic Laboratory, Federal University of Santa Maria, Santa Maria, RS, Brazil; 2 Pharmacology Graduate Program, Federal University of Santa Maria, Santa Maria, RS, Brazil; 3 Biochemical Toxicology Graduate Program, Federal University of Santa Maria, Santa Maria, RS, Brazil; 4 Laboratory of Molecular Biology, University of Western Santa Catarina, UNOESC, Chapecó, SC, Brazil; University of California Davis, United States of America

## Abstract

Methotrexate (MTX) is a folic acid antagonist used in high doses as an anti-cancer treatment and in low doses for the treatment of some autoimmune diseases. MTX use has been linked to oxidative imbalance, which may cause multi-organ toxicities that can be attenuated by antioxidant supplementation. Despite the oxidative effect of MTX, the influence of antioxidant gene polymorphisms on MTX toxicity is not well studied. Therefore, we analyzed here whether a genetic imbalance of the manganese-dependent superoxide dismutase (SOD2) gene could have some impact on the MTX cytotoxic response. An *in vitro* study using human peripheral blood mononuclear cells (PBMCs) obtained from carriers with different Ala16Val-SOD2 genotypes (AA, VV and AV) was carried out, and the effect on cell viability and proliferation was analyzed, as well as the effect on oxidative, inflammatory and apoptotic markers. AA-PBMCs that present higher SOD2 efficiencies were more resistance to high MTX doses (10 and 100 µM) than were the VV and AV genotypes. Both lipoperoxidation and ROS levels increased significantly in PBMCs exposed to MTX independent of Ala16Val-SOD2 genotypes, whereas increased protein carbonylation was observed only in PBMCs from V allele carriers. The AA-PBMCs exposed to MTX showed decreasing SOD2 activity, but a concomitant up regulation of the SOD2 gene was observed. A significant increase in glutathione peroxidase (GPX) levels was observed in all PBMCs exposed to MTX. However, this effect was more intense in AA-PBMCs. Caspase-8 and -3 levels were increased in cells exposed to MTX, but the modulation of these genes, as well as that of the Bax and Bcl-2 genes involved in the apoptosis pathway, presented a modulation that was dependent on the SOD2 genotype. MTX at a concentration of 10 µM also increased inflammatory cytokines (IL-1β, IL-6, TNFα and Igγ) and decreased the level of IL-10 anti-inflammatory cytokine, independent of SOD2 genetic background. The results suggest that potential pharmacogenetic effect on the cytotoxic response to MTX due differential redox status of cells carriers different SOD2 genotypes.

## Introduction

Methotrexate (MTX) is a drug that has been used since the 1950 s to treat a broad number of morbidities such as cancer and autoimmune diseases. The basis for its therapeutic efficacy is the inhibition of dihydrofolate reductase (DHFR), a key enzyme in folic acid (FA) metabolism [1]. At low concentrations, MTX has anti-inflammatory and/or immunosuppressive effects [2] related to the induction of lymphocyte apoptosis through oxidative stress and increasing caspase-3 levels [3], [4]. For this reason, it is the first-line therapy for the treatment of moderate to severe psoriasis and psoriatic arthritis all over the world [5].

In contrast, the continued use of MTX has being associated with oxidative imbalance, which may cause multi-organ toxicities, including hepato-, neuro-, lung- and nephrotoxicity and testicular damage [6]–[9]. Investigations suggest that oxidative stress caused by MTX involves decreasing in some antioxidant enzymes as glutathione peroxidase, glutathione reductase, catalase and superoxide dismutase, increasing of lipoperoxidation and reactive oxygen species (ROS) levels, as well as apoptosis induction [4], [10].

Despite the fact that the clinical response to MTX and its adverse effects exhibit marked interpatient variability indicating pharmacogenetic effects [11], the influence of antioxidant gene polymorphisms on MTX efficacy and toxicity is not well studied.

Human beings present genetic polymorphisms in antioxidant enzymes, which have an impact on cell oxidative metabolism and are associated with the risk of chronic diseases, such as the Ala16Val polymorphism in manganese-dependent superoxide dismutase (MnSOD or SOD2) [12]. This single nucleotide polymorphism (SNP) (rs4880) occurs in the target sequence of the SOD2 enzyme, where a valine to alanine substitution causes a SOD2 conformational change from beta-sheet to alpha helix, compromising the ability to neutralize O2- radicals. The α-helix SOD2 protein form produced by the A allele is related to a 30–40% increase in enzyme activity, whereas the V allele is related to reduced SOD2 enzyme efficiency [13].

Previous investigations have suggested that the AA genotype increases the susceptibility to develop some cancer types such as breast and prostate cancer, whereas other studies have associated the V allele with a higher risk of developing metabolic diseases such as obesity and hypercholesterolemia [14], [15]. In addition, the toxicogenetic and pharmacogenetic effects of the Ala16Val-SOD2 polymorphism were described to include the *in vitro* influence on the toxic response of human lymphocytes exposed to UV radiation [16] and methylmercury [17]. The differential response of peripheral blood mononuclear cells (PBMCs) to a clomiphene citrate, a gynecological drug with antioxidant activity, was also reported [18]. The investigation performed by Montano et al. [19] also described that the Ala16Val-SOD2 polymorphism could trigger PBMCs to produce different levels of proinflammatory cytokines when exposed to culture medium richest in glucose and/or insulin. In this case, the V allele presented higher levels of proinflammatory cytokines than did the A allele.

Therefore, we analyzed here whether the Ala16Val-SOD2 polymorphism could have some impact on the MTX cytotoxic response via an *in vitro* study using human peripheral blood mononuclear cells (PBMCs) from carriers of different Ala16Val-SOD2 genotypes.

Therefore, we analyzed the MTX effect at different concentrations on PBMCs viability and proliferation with different SOD2 genotypes. In addition, effect on redox metabolism, inflammatory and apoptosis pathway by MTX exposition was also investigated from evaluation of the levels of ROS, lipoperoxidation, protein carbonylation, genotoxicity, antioxidant enzymes activities, cytokines production and caspases (CASP) levels. Modulation of gene expression of antioxidant enzymes and some molecules involved with apoptosis pathway by MTX exposition was also determined.

## Materials and Methods

### General experimental design

An *in vitro* analysis was performed using human peripheral mononuclear cells (PBMCs) obtained from carriers of different SOD2 genotypes. The present research study was approved by the Ethics Committee of the UFSM (no 23081.015838/2011-10), and all blood cell donors signed a consent form.

### Reagents

MTX, thiazolyl blue tetrazolium bromide, 2′,7′-dichlorofluorescin diacetate, silver nitrate, and xanthine were obtained from Sigma-Aldrich (St. Louis, MO, USA). The Quant-iT TM Picogreen® dsDNA Assay Kit was obtained from Life-Technologies (Carlsbad, CA, USA). Reagents for cell culture including RPMI 1640 Medium, fetal bovine serum, penicillin/streptomycin and amphotericin were obtained from Sigma-Aldrich Reagents for molecular biology were as follows: Phusion Blood Direct PCR Kit (Thermo Scientific, Waltham, MA, USA), Trizol®, Dnase, SYBR® Green Master Mixes (Life-Technologies). The iScript cDNA synthesis kit was obtained from Bio-Rad (Berkeley, CA, USA). Caspase and cytokine immunoassays were performed using Quantikine® Colorimetric kits (R&D Systems, Minneapolis, MN, USA). The equipment used for ARMS-PCR (genotyping) and Q-PCR were Thermocycler (MaxygenII-Axygen, Union City, CA-USA) and StepOne Plus (Applied Biosystems, Foster City, CA, USA) instruments. Fluorimetric readings were obtained using a SpectraMax M2/M2e Multi-mode Plate Reader (Molecular Devices Corporation, Sunnyvale, CA, USA).

The effects of SOD2 genotype on cell viability, apoptosis induction, oxidative metabolism imbalance, genotoxicity and inflammatory cytokine levels were analyzed. To perform the experiments, we first collected blood samples and genotyped the Ala16Val-SOD2 gene single nucleotide polymorphism (SNP) of 120 healthy adult subjects. The SOD2 genotypes frequencies (AA = 22.8%, VV = 27.6% and AV = 48.7%) were in Hardy-Weinberg equilibrium that was calculated by chi-square goodness-of-fit statistical test. Further, some subjects with similar lifestyle profiles were invited to donate blood again and these samples were used to perform the *in vitro* assays. From this second blood donation, the PBMCs (1×10^5^ cells) were obtained and cultured in controlled conditions with and without MTX exposure (0, 0.1, 1, 10, and 100 µM). There are few studies involving MTX effects on PBMCs cells. Therefore we used a broad concentration range based in a previous investigation performed by Sakuma et al [20]. The cell viability was determined, and the effect of MTX on apoptosis, oxidative stress and inflammatory metabolism was evaluated and compared among all treatments. Apoptosis pathway induction by MTX was evaluated by quantifying caspase-8 and -3 levels. Caspase-8 is an apoptosis initiator molecule, which activates caspase-3, and represents a key point in the transmission of the proteolytic signal. The gene expression levels of these CASP were also determined. Because MTX oxidative stress could be related to mitochondrial damage that triggers apoptosis, we also analyzed the effect of MTX treatments on Bcl-2 and BAX gene modulation. These genes belong to the Bcl-2 family of gene proteins that is also involved in the apoptosis pathway. The effect of MTX on PBMC oxidative metabolism was evaluated by quantifying ROS, lipoperoxidation, protein carbonylation and genotoxicity levels. The levels of antioxidant enzymes [SOD1, SOD2, catalase (CAT), and glutathione peroxidase, (GPX)] were also determined, as were the effects of MTX on the gene expression of these enzymes. Because PBMCs produce important inflammatory cytokines modulated by MTX [21], the levels of interleukin-1 beta (IL-1β), interleukin-6 (IL-6), tumor necrosis factor alpha (TNFα), interferon gamma (IFNγ) and the anti-inflammatory cytokine interleukin-10 (IL-10) were measured and compared among PBMCs with different Ala16Val-SOD2 genotypes that had been exposed to MTX. The caspase-1 level was also determined because this intracellular cysteine protease is required for processing the IL-1 precursor into the mature and active form that can then be secreted from the cell [1]. All experiments were performed in triplicate, and the assays used to perform these analyses are described below.

### Ala16Val-SOD2 SNP genotyping

**Figure 1:**
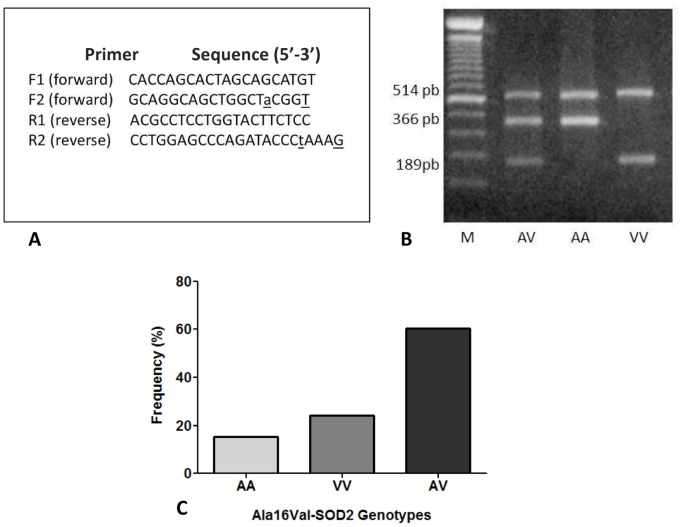
Ala16Val-SOD2 genotyping. (A) primers used to perform tetra-primer ARMS-PCR assay; (B) Gel electrophoresis showing different fragments used to identify the SOD2 genotypes; (C) Genotypic frequency distribution of AA, VV and AV genotypes in the 120 adult health samples subject that donate blood sample to perform the PBMCs *in vitro* assays.

To obtain PBMCs, the blood samples were first collected by venipuncture from 120 healthy adult subjects (26.4±7.3 years old) living in a Brazilian region (Rio Grande do Sul) without a history of diseases that are treated with MTX, non-smokers, not obese, no use of chronic medication or vitamin supplements, no previous cardiovascular medical history or hypertensive disorder, and no metabolic diseases or other morbidity that could affect the results. The Ala16Val-SOD2 genotyping was determined by polymerase chain reaction using a direct total blood cell sample and Tetra-Primer ARMS-PCR assay as described by Ruiz-Sanz et al. [22] with slight modifications. Briefly, two primer pairs were used to amplify and determined the genotype of a DNA fragment containing the Ala16Val SNP in the human SOD2 sequence. The 3′-end of the allele-specific primers is underlined. Underlined lowercase bases indicate the introduced mismatches. The PCR reaction was carried out in a total volume of 40 µL containing 20–40 ng of genomic DNA as the template, 0.5 µM of each primer, 100 µM of each dNTP, 1.25 mM of MgCl2, PCR buffer (20 mM Tris-HCl (pH 8.4), 50 mM KCl), 5% dimethyl sulfoxide (DMSO), and 1.25 Units of DNA polymerase. The PCR amplification was carried out with an initial denaturation at 94°C for 7 min, followed by 35 cycles of 60 s of denaturation at 94°C, 20 seconds of annealing at 60°C, and 30 s of extension at 72°C, and an additional 7 minutes of extension at 72°C at the end of the final cycle. A 20-µL aliquot of the PCR products was mixed with 6 µL of loading buffer and resolved by electrophoresis in a 1.5% agarose gel. This procedure resulted in three bands in heterozygotes (514, 366, and 189 bp) and two bands in homozygotes (Val/Val resulting in bands of 514 and 189 bp, and Ala/Ala resulting in bands of 514 and 366 bp) (Figure 1).

### PBMCs *in vitro* culture

From the subjects genotyped, a sub-group of 6–8 subjects per genotype with the Ala16Val-SOD2 genotype were invited to donate blood again in order to perform cell culture and *in vitro* assays involving MTX exposure as previously described in Montano et al. [19]. The 20 mL blood samples were collected by venipuncture using heparinized vials and then transferred to tubes with Ficoll histopaque (1∶1). The tubes were centrifuged for 30 min at 252×g and PBMCs were positioned in the interfase, PBMCs were centrifuged again (10 minutes at 2000 rpm) and transferred to culture medium containing 1 ml RPMI 1640 (GIBCO) with 10% fetal calf serum (FCS) and 1% penicillin/streptomycin. Culture tubes for each subject were prepared at a final concentration of 1×10^6^ cells/mL. The PBMC cultures were incubated at 37°C and 5% CO_2_ for 24 h before performing the experiments.

### Viability and Cell proliferation assays

Based on previous reports that low dose MTX exerts anti-inflammatory and immunosuppressive effects that induce apoptosis and oxidative stress [3], we exposed PBMCs from carriers of different Ala16Val-SOD2 genotypes to different MTX concentrations (0.1–100 µM). The effect of genotype on cell viability was analyzed after 24 hs of exposure and the effect on cell proliferation was assessed after 72 hs of exposure. Cell viability after 24 h of MTX exposition and cell proliferation after 72 h of MTX exposition was analyzed by the MTT (3-[4,5dimethylthiazol-2-yl]-2,5-diphenyltetrazolic bromide) reduction assay as described by Mosmann [23]. Briefly, treated cells were incubated for 4 h with MTT reagent. After the formazan salt was dissolved, the absorbance was measured at 570 nm. The cells were photographed before the addition of DMSO in order to observe the formazan crystals. The MTT assay was performed using a 96-well plate in three independent replications. The Trypan blue dye exclusion assay was also performed to confirm the MTX effect on PBMCs viability [24]. The results were expressed as a percentage of the untreated control values.

### 2′–7′-dichlorofluorescein diacetate (DCFDA) ROS production assay

The effect of 24 h of MTX exposure on the oxidative metabolism of PBMCs from carriers of different Ala16Val-SOD2 genotypes was evaluated for different oxidant and antioxidant variables. The ROS level was determined using the non-fluorescent cell permeating compound 2′–7′-dichlorofluorescein diacetate (DCFDA) assay. In this technique, the DCFDA is hydrolyzed by intracellular esterases to DCFH, which is trapped within the cell. This non-fluorescent molecule is then oxidized to fluorescent dichlorofluorescein (DCF) by cellular oxidants. After the designated treatment time, the cells were treated with DCFDA (10 µM) for 60 minutes at 37°C. In the assay, 1×10^5^ cells from each sample were used to measured ROS levels [18]. The fluorescence was measured at an excitation of 488 nm and an emission of 525 nm, and the results were expressed as picomoles/mL of 2′,7′-dichlorofluorescein (DCF) production from 2′,7′-dichlorofluorescin in reaction with ROS molecules present in the samples.

### Spectrophotometric assays

Oxidative stress indicators were measured in PBMCs samples. Thiobarbituric acid reactive substances (TBARS) were measured according to the modified method of Jentzsch et al. [25]. The carbonylation of serum proteins was determined by the Levine method with modifications [31]. Whole blood catalase (CAT) activity was determined by the method of Aebi [26] by measuring the rate of decomposition of H_2_O_2_ at 240 nm. Whole blood superoxide dismutase (SOD) activity was measured as described by McCord & Fridovich [27]. The Glutathione peroxidase activity was measured as Glutathione Peroxidase (GPX) as described by Flohe e Gunzler with modifications [28].

### DNA comet genotoxicity assay

The alkaline comet assay was performed as described by Singh et al. [29] in accordance with the general guidelines for use of the comet assay [30]. One hundred cells (50 cells from each of the two replicate slides) were selected and analyzed. Cells were visually scored according to tail length and received scores from 0 (no migration) to 4 (maximal migration). Therefore, the damage index for cells ranged from 0 (all cells with no migration) to 400 (all cells with maximal migration). The slides were analyzed under blind conditions by at least two different individuals.

### Caspase and cytokine immunoassays

The analyses of CASP- 8, -3, and -1 and cytokines IL-1β, IL-6, TNFα, Ig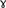, IL-10 were performed using the Quantikine Human Caspase Immunoassay to measure CASP in the cell culture supernatants, according to the manufacturer’s instructions. Briefly, all reagents and working standards were prepared and the excess microplate strips were removed. The assay diluent RD1W was added (50 mL) to each well. Further, 100 mL of standard control for our sample was added per well, after which the well was covered with the adhesive strip and incubated for 1.5 h at room temperature. Each well was aspirated and washed twice for a total of three washes. The antiserum of each molecule analyzed here was added to each well and covered with a new adhesive strip and incubated for 30 min at room temperature. The aspiration/wash step was repeated, and the caspase-1 conjugate (100 mL) was added to each well and incubated for 30 min at room temperature. The aspiration/wash step was repeated and 200 mL of substrate solution was added to each well and incubated for 20 min at room temperature. Finally, the 50 mL stop solution was added to each well and the optical density was determined within 30 min using a microplate reader set to 450 nm.

### mRNA expression analysis by quantitative QT-PCR assay

The expression levels of eight genes were measured by QT-PCR assay in PBMCs from carriers of different Ala16Val-SOD2 genotypes exposed to MTX: four genes belong to oxidative metabolism [superoxide dismutase genes (SOD1, SOD2), catalase (CAT) and glutathione peroxidase (GPX)]. The gene expression of some proteins involved in the apoptosis cascade, initiator caspase-8 (CASP 8) and effector caspase-3 (CASP 3) were also evaluated, as was the pro-apoptotic Bcl-2-associated X protein (BAX) gene, which has been shown to be involved in p53-mediated apoptosis.

Total RNA was isolated using TRIzol reagent. RNA yields were measured using a Nanodrop 2000 spectrophotometer. First strand cDNA was synthesized from total RNA (2 µg) using a First Strand cDNA Synthesis Kit and oligo dT primers. Q-PCR was performed in a 10 µl reaction that contained 0.5 µl of the cDNA and 1×KAPA SYBR® FAST Universal qPCR Master Mix (Kapa Biosystems, Woburn, MA, USA) using the following PCR parameters: 95°C for 3 min followed by 40 cycles of 95°C for 10 s, 60°C for 30 s followed by a melt curve of 65°C to 95°C in 0.5°C increments for 5 s. The expression level of beta-actin was used as an internal control. The relative expression was calculated using the comparative Ct and was expressed as the fold expression compared to the control. The specific primer pairs of antioxidant gene enzymes are presented used in this study were: SOD1 Forward GCACACTGGTGGTCCATGAA and Reverse ACACCACAAGCCAAACGACTT; SOD2 Forward- 5′GCCCTGGAACCTCACATCAA3′ and Reverse- GGTACTTCTCCTCGGTGACGTT; CAT = Forward- GATAGCCTT CGACCCAAGCA and Reverse- ATGGCGGTGAGTGTCAGGAT; GPX = Forward- GGTTTTCATCTATGAGGGTGTTTCC and Reverse- GCCTTGGT CTGGCAGAGACT; BAX = Forward- CCCTTTTCTACTTTGCCAGCAA and Reverse- CCCGGAGGAAGTCCAATGT; Bcl-2 = Forward- GAGGATT GTGGCCTTCTTTGAGT; Reverse- AGTCATCCACAGGGCGATGT; CASP3 = Forward- TTTGAGCCTGAGCAGAGACATG and Reverse- TACCAGT GCGTATGGAGAAATGG; CASP 8 = Forward- AGGAGCTGCTCTTCCGAATT and Reverse- CCCTGCCTGGTGTCTGAAGT.

### Statistical analysis

All analyses were carried out using the Graph Pad Prism 5 software, and the results were expressed as the mean ± standard deviation (SD). The comparison of all PBMC samples from different Ala16Val-SOD2 donors treated with and without MTX was performed using the two-way analysis of variance followed by a *post hoc* Tukey’s test. All p values were two-tailed. The alpha value was set to <0.05 to determine statistical relevance.

## Results

**Figure 2:**
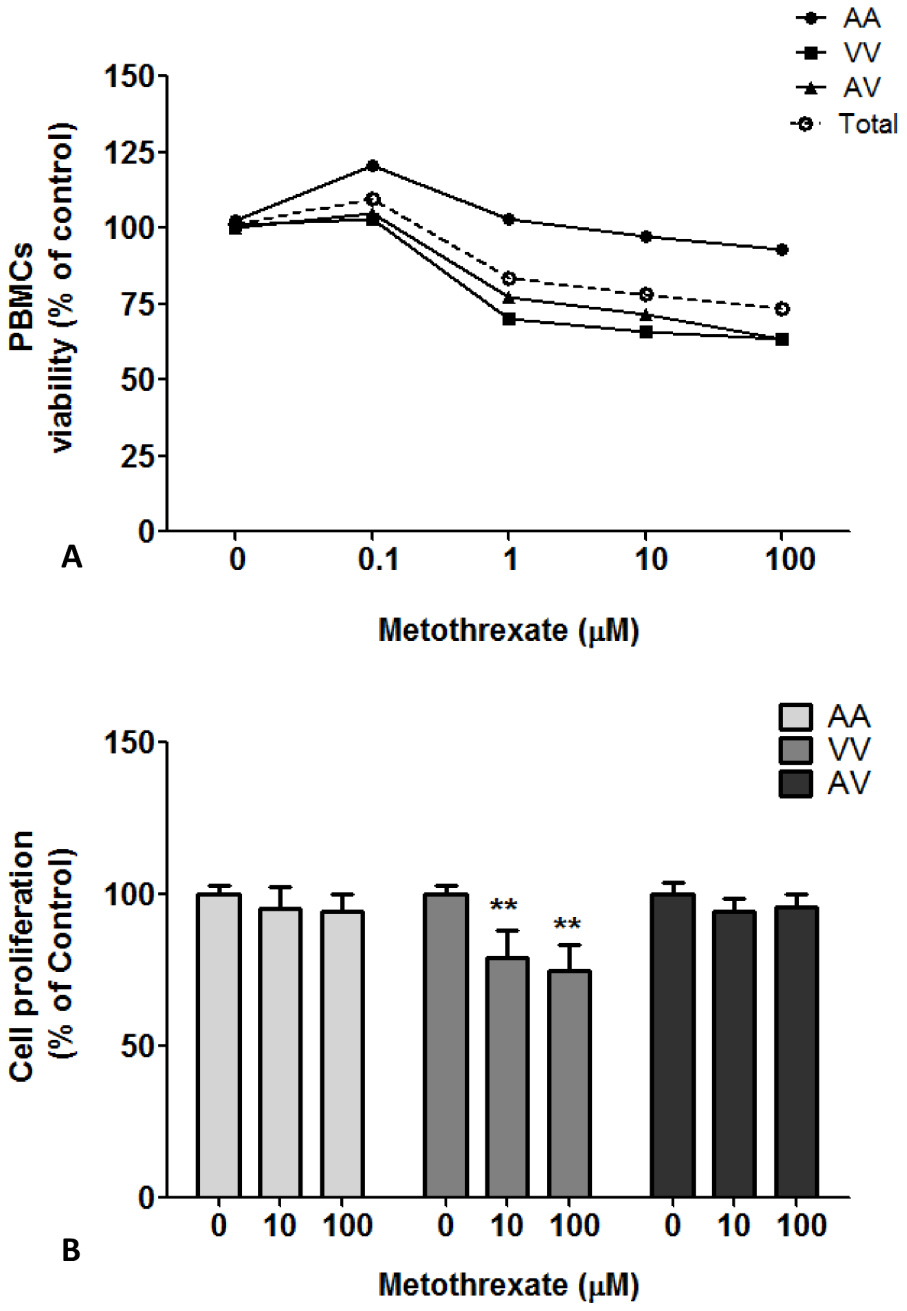
MTX effect on PBMCs carrier's different Ala16Val-SOD2 genotypes. (A) cell viability at different MTX concentrations evaluated after 24 h of MTX exposition; (B) cell proliferation at different MTX concentrations evaluated after 72 h of MTX exposition. **p<0.01 was determined by two-way analysis of variance followed by Bonferroni *post hoc* test. Viability and cell proliferation were evaluated by MTT assay.

The MTX exposure caused significant cytotoxicity from 1 µM concentration in human PBMCs. However, this effect was significantly influenced by the Ala16Val-SOD2 SNP (Figure 2A). PBMCs from carriers with the V allele (VV and AV) exhibited decreased viability when exposed to MTX at 1, 10 and 100 µM concentrations, whereas AA-PBMC viability was not affected by these treatments. These results were confirmed by trypan assay.

Based on these results, a second analysis was performed to evaluate the prolonged MTX effect at 10 and 100 µM concentrations on PBMC proliferation. Again, the cell response was influenced by the Ala16Val-SOD2 SNP. As seen in Figure 2B, MTX did not influence the cell proliferation of PBMCs from A-carriers (AA and AV). The VV-PBMCs treated with MTX showed significant decreases in proliferation rate when compared to the untreated control group. However, the influence on VV-cell proliferation was similar at the 10 and 100 µM MTX concentrations.

The influence of MTX on PBMC oxidative metabolism after 24 h of exposure was then analyzed, and the results are presented in Table 1. Lipoperoxidation as well as ROS levels increased significantly in PBMCs exposed to MTX independent of Ala16Val-SOD2 genotype. However, the effect on lipoperoxidation was not dependent on MTX concentration (10 and 100 µM), whereas the increase in ROS levels only occurred at the higher MTX dose tested here (100 µM). On the other hand, protein carbonylation was not affected in AA-PBMCs, whereas AV and VV PBMCs presented an increase in this oxidative parameter. However, the effect of MTX on protein carbonylation was dose-dependent only in VV-PBMCs.

**Table 1:** Comparison of oxidative metabolism variables of peripheral blood mononuclear cells (PBMCs) carrier’s different Ala16Val-SOD2 genotypes exposed to Methotrexate.

Variables	MTX (µM)	Ala16Val-SOD2 Genotypes		
		AA	VV	AV
		Mean ± sd	Mean ± sd	Mean ± sd
TBARS (mmol MDA/mg protein)	0	3.210±0.014^a^	3.805±0.020^a^	3.390±0.031^a^
	10	5.165±0.274^b^	5.307±0.08^b^	6.290±0.215^b^
	100	5.008±0.301^b^	4.948±0.176^b^	6.000±0,412^b^
Protein carbonylation (mmol/mg protein)	0	0.226±0.023^a^	0.201±0.023^a^	0.195±0.024^a^
	10	0.267±0.022^a^	0.292±0.030^b^	0.339±0.079^b^
	100	0.213±0.015^a^	0.329±0.035^c^	0.306±0.04^b^
ROS (DCF picomoles/mL)	0	3104±177^a^	2680±199^a^	2855±330^a^
	10	3704±193^a^	2829±132^a^	2693±261^a^
	100	5632±191^b^	7311±269^b^	4999±509^b^
SOD1 (UMnOD/mg protein)	0	0.747±0.09^a^	0. 610±0.04^a^	0.654±0.04^a^
	10	0.507±0.05^b^	0.423±0.05^b^	0.456±0.06^b^
	100	0.498±0.06^b^	0.411±0.04^b^	0.432±0.05^b^
SOD2 (UMnOD/mg protein)	0	2.170±0.190^a^	0.728±0.05a^a^	1.050±0.160^a^
	10	1.577±0.125^b^	0.756±0.05^a^	0.870±0.270^a^
	100	1.170±0.04^b^	0.580±0.01^b^	0.960±0.110^a^
Catalase (K/mg protein)	0	0.050±0.004^a^	0.043±0.008^a^	0.033±0.008^a^
	10	0.024±0.003^b^	0.037±0.002^b^	0.041±0.006^a^
	100	0.027±0.003^b^	0.041±0.018^a^	0.039±0004^a^
GPX (U/mL)	0	4.01±0.82^a^	11.04±2.02^a^	11.75±3.04^a^
	10	12.96±2.02^b^	16.73±3.04^b^	46.86±4.03^b^
	100	20.53±3.50^c^	22.23±3.06^c^	36.39±3.02^c^

MTX = methotrexate; sd = standard deviation; Different letters (a, b, c) indicate significant differences among each MTX treatment determined by analysis of variance followed by Tukey's post hoc test at p<0.05.

**Figure 3:**
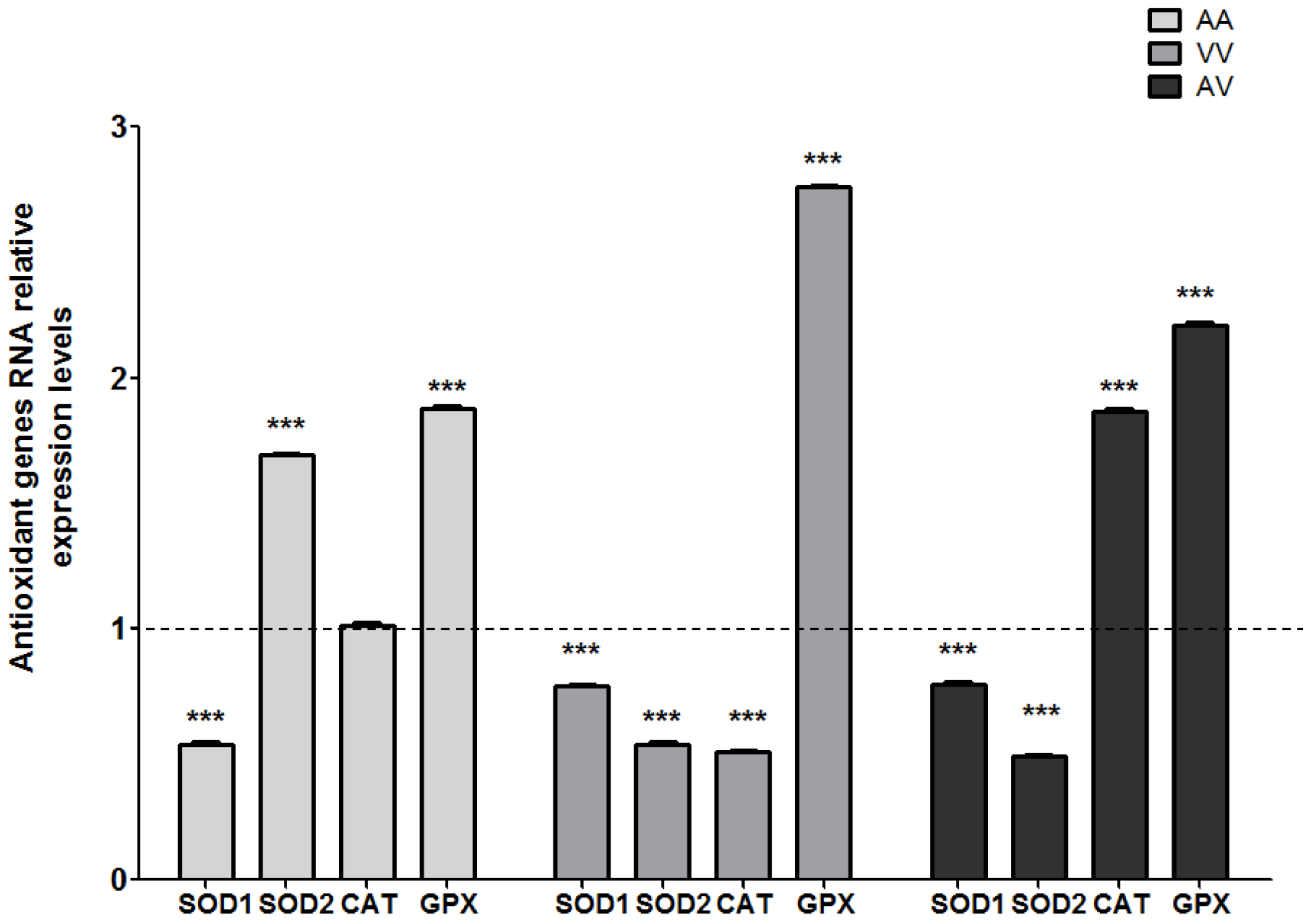
MTX (10 µM) effect on antioxidant SOD1, SOD2, CAT and GPX genes expression. The dashed line on the value 1 indicate that each untreated control group of PBMCs carrier's different Ala16Val-SOD2 genotypes was used as reference to calculated the relative mRNA expression of the antioxidant enzymes. **p≤0.01 and *** p≤0.001 were determined by two-way analysis of variance followed by Bonferroni *post hoc* test.

The effects of MTX on antioxidant enzyme activity and gene expression were evaluated. However, considering the results obtained in the analysis of antioxidant activity, the effect on gene expression was evaluated only in cells exposed to 10 µM MTX (Table 1, Figure 3). SOD1 was strongly affected by MTX exposure, resulting in decreased enzyme activity and gene expression independent of the Ala16Val-SOD2 genotype.

In contrast, the SOD2 activity and the gene expression were influenced by the Ala16Val-SOD2 SNP. The AA-PBMCs exposed to MTX showed decreasing SOD2 activity. However, SOD2 gene expression significantly was upregulated in the cells exposed to MTX. The VV-PMCs presented a decrease in SOD2 activity only when exposed to the higher MTX concentration (100 µM). Unlike the AA-PBMCs, these cells presented SOD2 gene down regulation when compared to the control group. Despite the fact that AV-PBMCs also presented SOD2 gene down regulation, the SOD2 activity was maintained in cells treated with MTX.

Catalase activity decreased only in AA-PBMCs cells exposed to MTX. However, these cells did not demonstrate any effect on catalase gene expression. The PBMCs from carriers of the A allele (AA and AV) did not show a decrease in catalase levels, but the effect on catalase gene expression was evident. Whereas, VV-PBMCs exposed to MTX exhibited downregulated catalase gene expression, heterozygous cells demonstrated catalase upregulation.

After 24 hs of MTX exposure, PBMCs presented high levels of GPX enzyme when exposed to MTX drug, independent of genetic background. The GPX gene was strongly upregulated in cells treated with MTX when compared to untreated control cells.

**Figure 4:**
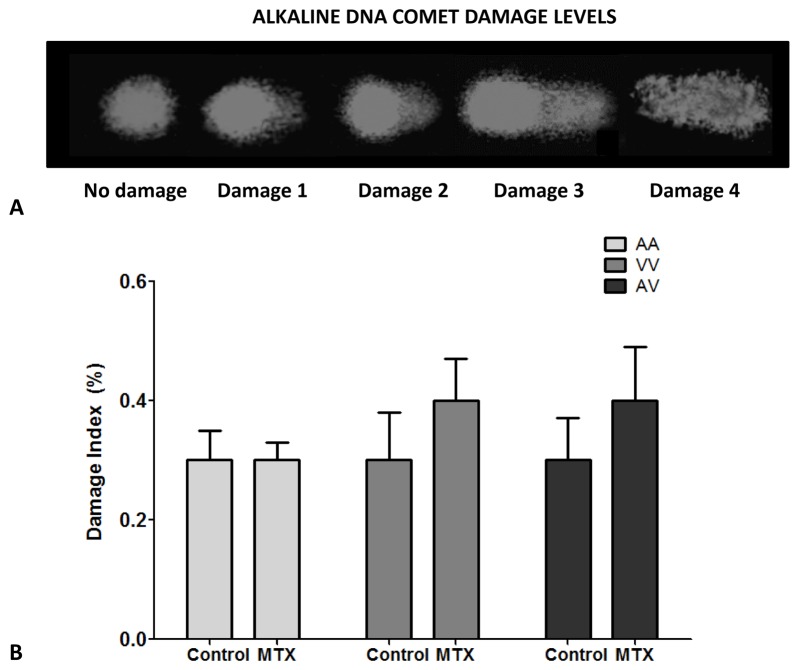
DNA damage to MTX exposition in PBMCs from subjects with different Ala16Val-SOD2 genotypes. (A) alkaline DNA comet assay showing nucleus without damage, and the nucleus with different damage levels; (B) Index damage of cells no-exposed and exposed to MTX 10 µM. No significant differences were observed between treatments.

The potential genotoxic effect in survival cells exposed to MTX at 10 µM concentration was evaluated. The results presented in Figure 4 show no statistically significant differences in terms of DNA damage in PBMCs obtained from carriers of different Ala16Val-SOD2 genotypes.

The cytokines involved in inflammatory response were also determined in PBMCs from carriers of different Ala16Val-SOD2 genotypes exposed to MTX. The results described in Table 2 show that 24 h of 10 µM MTX exposure significantly increase levels of inflammatory cytokines (IL-1β, IL-6, TNFα and Igγ) and significantly decrease IL-10, an anti-inflammatory cytokine. These results were similar in all PBMCs independent of Ala16Val-SOD2 genotype.

**Table 2:** Comparison of cytokines involved in immune response of peripheral blood mononuclear cells (PBMCs) carrier’s different Ala16Val-SOD2 genotypes exposed to Methotrexate.

Variables	MTX (µM)	Ala16Val-SOD2 Genotypes		
		AA	VV	AV
		Mean ± sd	Mean ± sd	Mean ± sd
Interleukin 1β (pg/mL)	0	46.3±5.9^a^	51.7±2.0^a^	44.6±3.7^a^
	10	210.3±16.6^b^	346.1±12.6^b^	321.1±6.9^b^
Interleukin 6 (pg/mL)	0	58.0±5.9^a^	56.3±7.8^a^	60.7±4.1^a^
	10	251.9±43.1^b^	472.8±108^b^	433.3±8.6^b^
TNFα (pg/mL)	0	86.0±4.3^a^	86.3±7.6^a^	88.6±2.14^a^
	10	278.7±57.5^b^	494.9±7^b^	449.3±60.9^b^
Igγ (pg/mL)	0	103.2±9.4^a^	100.7±7.6^a^	108.6±6.8^a^
	10	351.2±68.1^b^	668.2±25.9^b^	589.7±9.4^b^
Interleukin 10 (pg/mL)	0	86.7±4.7^a^	90±4.2^a^	90.0±6.1^a^
	10	68.3±16.3^b^	54.6±9.6^b^	46.9±2.4^b^

MTX = methotrexate; sd = standard deviation; Different letters (a, b, c) indicate significant differences among each MTX treatment determined by analysis of variance followed by Tukey's post hoc test at p<0.05.

**Figure 5:**
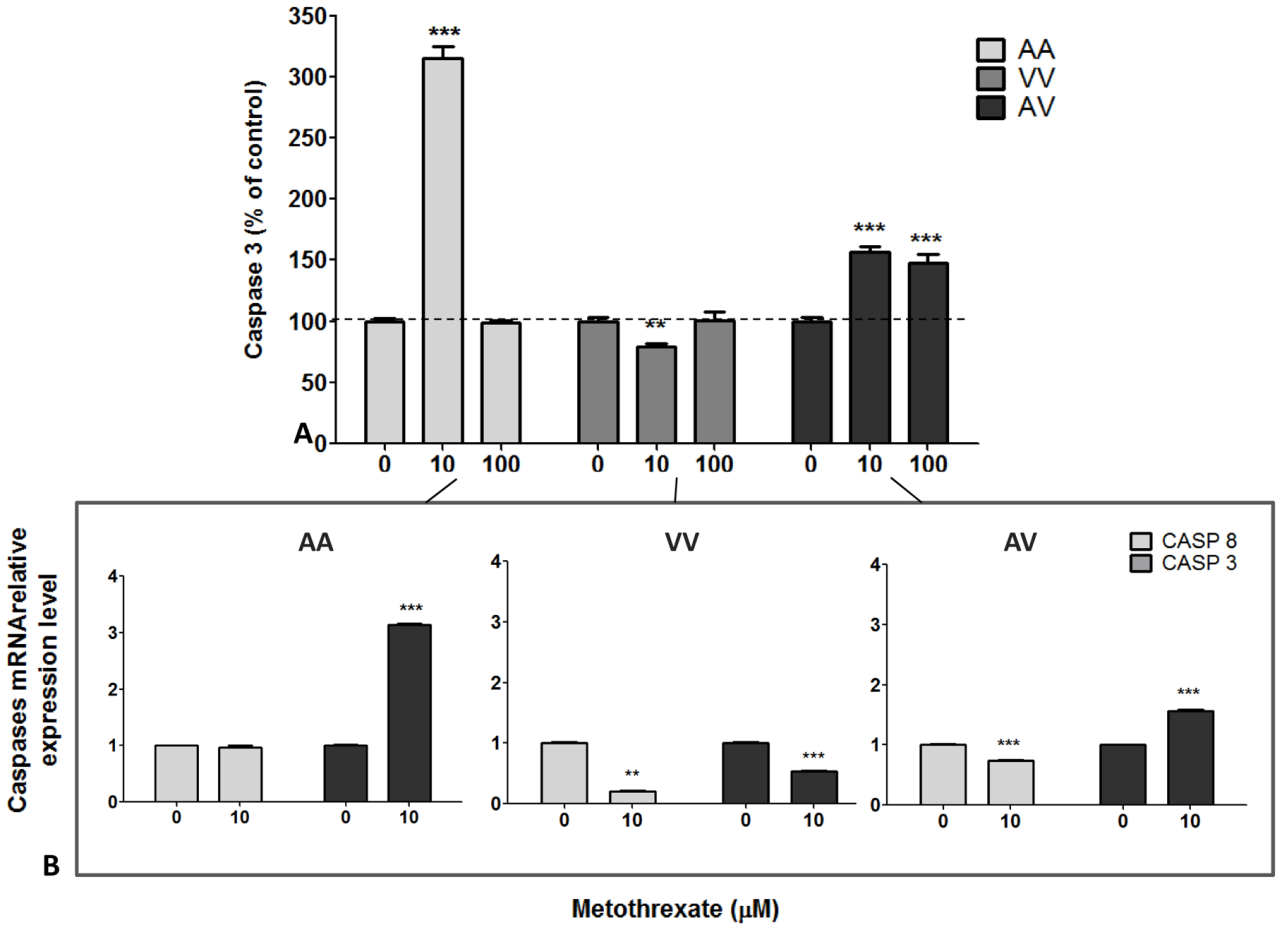
MTX (10 µM) effect on (A) CASP 8 and 3 protein levels determined by immunoassay tests and (B) respective gene expression in PBMCs carriers different Ala16Val-SOD2 genotypes. The dashed line on the value 1 indicate that each untreated control group of PBMCs carrier's different Ala16Val-SOD2 genotypes was used as reference to calculated the relative mRNA expression of the antioxidant enzymes. **p≤0.01 and *** p≤0.001 were determined by two-way analysis of variance followed by Bonferroni *post hoc* test.

The effect of MTX on PBMC apoptosis was evaluated by determining CASP-3 and -8 gene and protein levels. The results presented in Figure 5 show increased CASP-1, -3 and -8 levels in cells exposed to 10 µM MTX. This result was independent of the Ala16Val-SOD2 SNP. However, the gene expression analysis showed significant differences among PBMCs with different genotypes. VV-PBMCs exposed to MTX presented the down regulation of both CASP genes (-8 and -3) when compared to the control group. In contrast, casp-8 was upregulated in AA-PBMCs. The heterozygous genotype showed an intermediary pattern of casp gene expression, where casp-8 was downregulated similar to the VV genotype and casp-3 was upregulated similar to the AA genotype.

**Figure 6:**
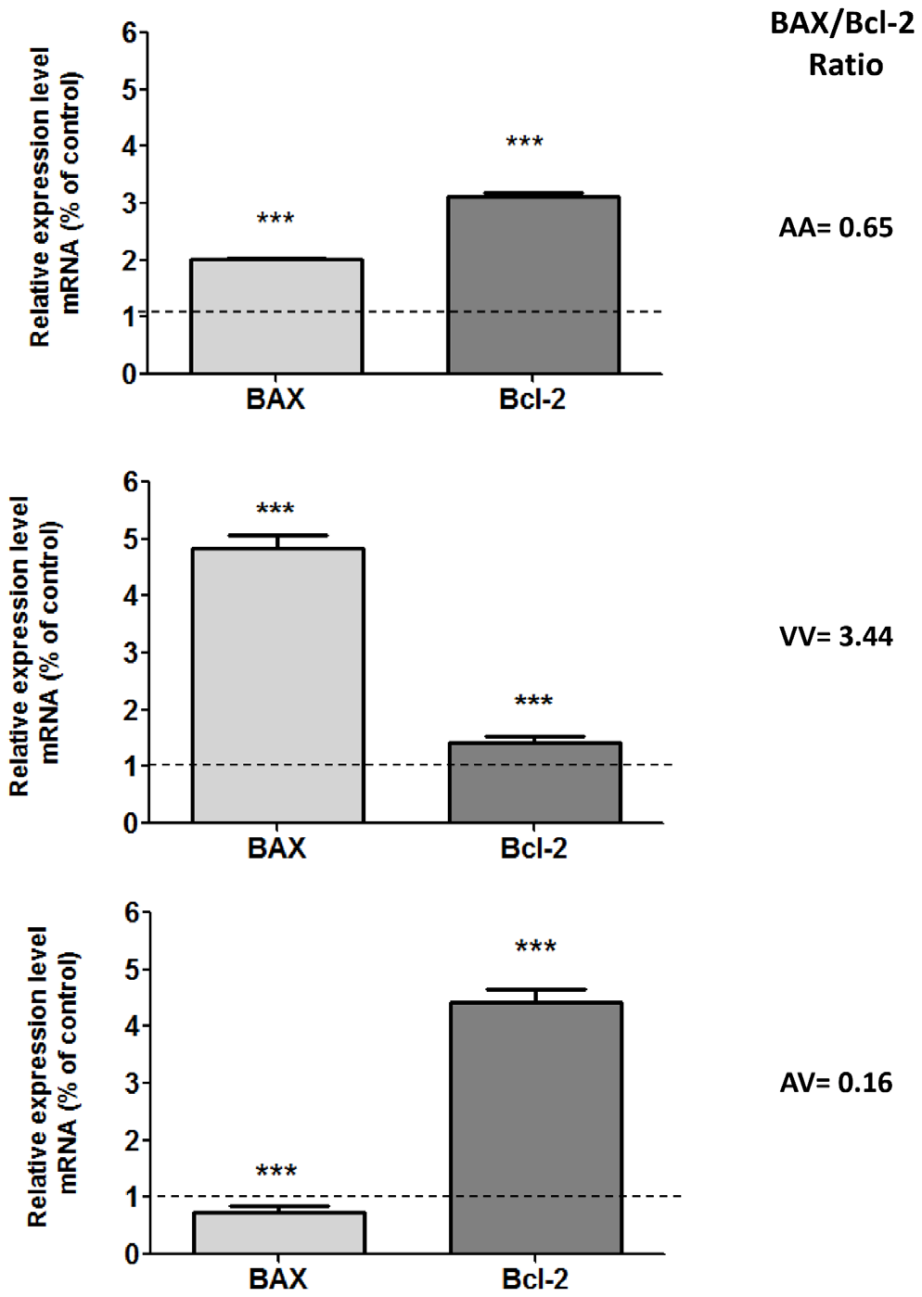
MTX (10 µM) effect on BAX and Bcl-2 gene expression of PBMCs carriers different Ala16Val-SOD2 genotypes. The dashed line on the value 1 indicate that each untreated control group of PBMCs carrier's different Ala16Val-SOD2 genotypes was used as reference to calculated the relative mRNA expression of the antioxidant enzymes. **p≤0.01 and *** p≤0.001 were determined by two-way analysis of variance followed by Bonferroni *post hoc* test. The ratio between expressions of BAX/Bcl-2 was also calculated. Values ≥1 indicate proapoptotic tendency and<indicate antiapoptotic tendency.

The effect of MTX on BAX and Bcl-2 gene expression was also evaluated, and the results showed an imbalance between these genes that was influenced by the SOD2 genetic background (Figure 6). The BAX gene was down regulated only in AV-PBMCs, whereas the BAX gene was upregulated in homozygous genotypes. The Bcl-2 gene was strongly upregulated in AA and AV-PBMCs and slightly upregulated in VV-PBMCs. Considering that the BAX/Bcl-2 balance defines the proapoptotic and antiapoptotic cell state, we also calculated the BAX/Bcl-2 ratio. The results showed an antiapoptotic ratio in AA and AV-PBMCs and a proapoptotic ratio in VV-PBMCs after 24 h of 10 µM MTX exposure.

## Discussion

The present study, confirmed that the cytotoxic effect of MTX, a commonly used anti-inflammatory, antiproliferative, and immunosuppressive drug, on human PBMCs involves an acute imbalance of cell oxidative and inflammatory metabolism and triggers apoptosis [14], [10], [11], [25]. However, our results showed that this effect was directly influenced by genetic background related to oxidative metabolism, specifically by the Ala16Val-SOD2 SNP.

The PBMCs obtained from healthy adult carriers of different Ala16Val-SOD2 genotypes showed a differential response to MTX in terms of cell viability and proliferation. Whereas AA showed some resistance to the immunosuppressive effect caused by MTX, presenting similar viability and cell proliferation levels than untreated cells, VV was more susceptible and presented reduced viability and cell proliferation. On the other hand, the heterozygous genotype (AV) showed an intermediary response to MTX exposure, observed as decreased cell viability as observed in VV-PBMCs and the maintenance of cell proliferation as observed in AA-PBMCs.

These results suggest that the SOD2 balance could play some pharmacogenetic or toxicogenetic role in the cellular MTX response. A robust number of studies have described that MTX at high concentrations causes oxidative stress in some types of cells. This observation was noted in the *in vitro* investigation performed by Chibbers et al. [31] which showed that MTX alone or in combination with Cu (II) was able to inhibit scavengers of ROS and exhibit pro-oxidant action. The oxidative imbalance in the Jurkat T lymphocytic line exposed *in vitro* to MTX was also described and related to apoptosis events [25]. Another investigation showed that MTX-induced oxidative stress in liver mitochondria caused a significant increase in mitochondrial lipid peroxidation, protein carbonyl content, superoxide radical (O_2_^−•^) generation and also affected the mitochondrial thiol profile [32]. MTX concentrations ≥10 µM also cause reduced antioxidant enzyme levels including superoxide dismutase, catalase and glutathione levels.

In the present study, the effect of MTX on AA-PBMC viability and cell proliferation was less intense than that observed in V allele carriers, indicating that MTX toxicity could be influenced by oxidative metabolism involving SOD2 modulation. This suggestion was confirmed by the analysis of potential causal mechanisms associated with the differential MTX response of PBMC carriers with different Ala16Val-SOD2 genotypes. The results showed that some variables presented a similar response to MTX independent of genetic background, including the increase of lipoperoxidation, inflammatory cytokines and apoptotic CASPs (-8 and -3) and GPX activity and gene expression, and the decrease in SOD1 activity and gene expression and IL-10, an anti-inflammatory cytokine.

However, in contrast to that observed in cytokine modulation some oxidative and apoptotic markers were differentially modulated in the PBMCs from carriers with different Ala16Val-SOD2 genotypes.

Considering the oxidative metabolism, we observed that AA-PBMCs did not show an increase in protein carbonylation, as occurred in the VV and AV genotypes. These cells also showed a decrease in SOD2 activity although we also observed the up regulation of this gene and an important increase in GPX enzyme levels and gene expression. The AA cells treated with MTX showed an approximately four-fold increase in GPX enzyme levels when compared to the untreated control group. Despite the fact that the VV and AV cells also presented increased GPX activity when treated with MTX, this effect was not as intense. The AA-PBMCs exposed to MTX also presented a decrease in catalase activity despite the fact that the gene expression of this enzyme maintained levels similar to those observed in the control group.

The AA genotype has been associated with high efficiency to dismutate O_2_^−•^ ions in hydrogen peroxide (H_2_O_2_) [12], and this property could be responsible for decreasing the oxidative stress caused by high levels of MTX and the subsequent decrease in apoptosis events observed in PBMCs from V allele carriers. In addition to the greater efficiency of AA-PBMCs in the dismutation of superoxide into hydrogen peroxide, these cells presented a significant increase in GPX levels when exposed to MTX, which probably offered some protection against toxic effects caused by hydrogen peroxide produced by superoxide dismutation.

The relevance of the AA genotype’s efficiency in controlling O_2_^−•^ and H_2_O_2_ levels in the cells exposed to MTX could have a consequence as a superior control mechanism for protein carbonylation production. Both O_2_^−•^ and H_2_O_2_ are capable of altering proteins chemically, thereby influencing their function. The main protein modifications originating from such an increase in oxidative stress comprise direct oxidation, namely that of amino acids with a thiol group such as cysteine, oxidative glycation, and carbonylation. In this context, it is remarkable that oxidative protein carbonylation, apparently the most frequent type of protein modification in response to oxidative stress, is thought to be irreversible and destined only to induce protein degradation in a nonspecific manner [33].

Therefore, events that prevent the production of high levels of protein carbonylation, including SOD2 efficiency and increased GPX activity and gene expression observed in AA-PBMCs exposed to MTX could explain why this genotype has protective effects against apoptosis events caused by MTX exposure.

On the other hand, VV cells exposed to MTX presented higher lipoperoxidation, protein carbonylation and ROS levels than untreated cells (Table 1). These results indicated increase in H_2_O_2_ levels triggered by MTX. However, opposite effects on main enzymes that catalyze H_2_O_2_ were also observed, since VV cells exposed to MTX showed lower CAT activity and higher GPX activity. These differences appear to be triggered by differential mRNA regulation of these enzymes (down regulation of CAT and upregulation of GPX genes). Considering the role of H_2_O_2_ in different signaling cellular cascades, this molecule is under sophisticated fine control of several antioxidant enzymatic molecules. However, despite CAT to be frequently used by cells to rapidly catalysis of H_2_O_2_ into less reactive gaseous oxygen and water molecules, the predominant scavengers of H_2_O_2_ in normal mammalian cells are likely other molecules as GPX and peroxiredoxins [34]. Our results suggest that in the presence of prooxidant molecules as MTX, the control of H_2_O_2_ production is directly influenced by efficiency of SOD2 enzyme. This suggestion can be partially corroborated by a previous study performed by Paludo et al. [35] suggested that Ala16Val-SOD2 SNP actively participates in the regulation of cellular redox environment involving H_2_O_2_ catalysis However the nature of the differential regulation of enzymes involving in H_2_O_2_ catalysis need to be clarified from complementary studies using antagonist and agonist molecules of SOD2 enzyme.

The heterozygous genotype (AV) showed an intermediary response to MTX exposition when compared to homozygous genotypes (AA and VV) or, sometimes the response was similar to AA genotype or to VV genotype.

The lesser effect of MTX on AA-PBMCs can also be observed when the expression of BAX and Bcl-2 genes was analyzed. In many systems, members of the bcl-2 family modulate apoptosis, with the BAX/Bcl-2 ratio serving as a rheostat with which to determine the susceptibility to apoptosis. Bcl-2 protein is able to repress a number of apoptotic death programs. Therefore, Bcl-2 is specifically considered an important anti-apoptotic protein and is, therefore, classified as an oncogene. In contrast, overexpressed BAX accelerates apoptotic death [36]. In healthy cells, BAX protein is largely found in the cytosol. However, upon initiation of apoptotic signaling, Bax undergoes a conformational shift and becomes organelle membrane-associated, in particular with the mitochondrial membrane. The main BAX effect involves the induction of opening of the mitochondrial voltage-dependent anion channel that results in the release of pro-apoptotic factors from the mitochondria, leading to the activation of CASPs [37].

The results showed differential Bax/Bcl-2 ratio gene expression in PBMCs from carriers of different Ala16Val-SOD2 genotypes exposed to MTX. Whereas the BAX/Bcl-2 ratio was below one in AA- and AV-PBMCs indicating a tendency to antiapoptotic events, VV-PBMCs showed a higher Bax/Bcl-2 ratio indicating the maintenance of cellular apoptosis. Therefore, these results confirmed the influence of Ala16Val-SOD2 on PBMC susceptibility to MTX exposure.

Another important result described here was the massive effect of MTX at the 10 µM concentration on human PBMC inflammatory cytokine levels. As previously mentioned, cells treated with MTX showed higher levels of IL-1β, IL-6, TNFα and Igγ. A reduction of IL-10, an anti-inflammatory cytokine, was also observed in cells exposed to MTX. Some of the results on the MTX inflammatory effect of PBMCs exposed to high levels of this drug have being described in previous studies performed in experimental models. For example, nephrotoxicity in rats induced by high doses of MTX increase the TNFα cytokine levels [38]. Rats with hepatorenal oxidative injury induced by high doses of MTX also showed increasing TNFα and IL-1β levels when compared to the untreated control group [39]. The number of studies analyzing the effect of MTX on IL-6 and Igγ is much lower [24], [40]. Therefore, to the best of our knowledge, the study of the concomitant effect of MTX on IL-1β, CASP 1, IL-6, TNFα, Igγ and IL-10 has not been previously published in the literature.

The clinical relevance of the data presented here could be related to the lower cytotoxic effect observed in AA-PBMCs exposed to MTX. This effect could be desirable in cancer patients undergoing chemotherapy using MTX. For example, neurocognitive sequelae associated with oxidative stress have been described in pediatric lymphoblastic leukemia patients treated with MTX [38], as have hepatotoxic and nephrotoxic effects [10], [11], [12].

However, the immunosuppressive activities of MTX have been studied in the context of cell proliferation and recruitment, and an inverse effect of MTX at low concentrations on some inflammatory cytokines is well established. Low MTX doses are able to reduce some important cytokines as TNFα that are elevated in autoimmune diseases such as rheumatoid arthritis [41]. Taking into account the results described here and published in the literature, we can suggest that MTX presents an important dose-dependent modulation of immune cytokines in PBMCs. This effect does not seem to be directly influenced by oxidative metabolism involving SOD2 activity because the results found here were independent of the Ala16Val-SOD2 SNP.

In conclusion, despite the methodological limitations related to *in vitro* experimental studies including the limited number of subjects used to obtain PBMCs with different SOD2 genotypes, the results described here suggest that the differential modulation of the cell’s O_2_^−•^ and H_2_O_2_ balance is genetically determined by SOD2 gene variation, which could influence the MTX cytotoxic effect.

These results are in consonance with previous studies describing the toxicogenetic and pharmacogenetic effects of the Ala16Val-SOD2 SNP on PBMCs exposed to xenobiotic molecules [21], [22]. Another important effect observed in this study was the MTX effect on cytokines involved in the inflammatory response, but this result seems to not be influenced by SOD2 metabolism.
